# Psychological and Clinical Challenges in the Management of Type 1 Diabetes during Adolescence: A Narrative Review

**DOI:** 10.3390/children11091085

**Published:** 2024-09-04

**Authors:** Bruno Bombaci, Arianna Torre, Alessandro Longo, Maria Pecoraro, Mattia Papa, Lacrima Sorrenti, Mariarosaria La Rocca, Fortunato Lombardo, Giuseppina Salzano

**Affiliations:** 1 Department of Human Pathology in Adult and Developmental Age “Gaetano Barresi”, University of Messina, 98122 Messina, Italy; ariannatorre95@gmail.com (A.T.); danila.pec296@gmail.com (M.P.); mattiapapa@msn.com (M.P.); lacrima.sorrenti@libero.it (L.S.); mrlarocca21@gmail.com (M.L.R.); fortunato.lombardo@unime.it (F.L.); gsalzano@unime.it (G.S.); 2 Department of Clinical and Experimental Medicine, University of Messina, 98122 Messina, Italy; ale.longo.92@gmail.com

**Keywords:** anxiety, automated insulin delivery, continuous glucose monitoring, depression, diabulimia, eating disorders, pediatrics, time in range, psychological, substance abuse

## Abstract

Adolescence, a critical period of physical and psychological development, presents unique challenges in type 1 diabetes (T1D) management due to endocrinological changes, reduced therapeutic adherence, and elevated susceptibility to psychological issues such as depression, anxiety, and eating disorders. This narrative review explores the impact of psychological and behavioral factors on glycemic control in adolescents with T1D. We examine the prevalence and influence of mental health disorders, lifestyle factors, harmful behaviors, and social dynamics on diabetes management and glycemic outcomes. Strategies for improving metabolic control are also reviewed, including cognitive behavioral therapy, technological devices, and educational interventions. The importance of tailored psychological support, family involvement, and targeted interventions to improve adherence to treatment and glycemic control in adolescents with T1D should be emphasized.

## 1. Introduction

Type 1 diabetes (T1D) is a chronic metabolic disorder caused by the autoimmune destruction of pancreatic β-cells, leading to the lack of endogenous insulin production and requiring lifelong insulin replacement therapy [1]. T1D is the most common form of diabetes in pediatric age, and one of the main chronic illnesses of childhood, accounting for 651,700 children and adolescents worldwide affected by this condition in 2021, and a predicted prevalence of 13.5–17.4 million in 2040, with a particular rise among low- and lower-middle-income countries [2].

The management of T1D primarily involves intensive insulin regimens, which consist of multiple daily injections or, given the recent advancements in diabetes technology, insulin pump therapy. However, effective insulin treatment also requires ongoing education, regular physical activity—which is crucial for reducing cardiometabolic risk—and adherence to a balanced diet [3,4]. Dietary regimen should ensure the optimal intake of all macronutrients, with carbohydrates accounting for approximately 40–50% of total energy intake, fat for less than 35% (with saturated fats under 10%), and protein for 15–25%. Greater flexibility in meal planning is achievable when insulin dosages are adjusted according to carbohydrate intake, positively impacting both glycemic control and quality of life [5].

Managing diabetes can be particularly challenging during adolescence, a period marked by rapid physical, sexual, and neurological maturation. This developmental stage often brings psychological and behavioral issues that can compromise glycemic control [6].

Adolescents are more susceptible to psychological issues compared to other life stages, experiencing a broad spectrum of emotional disorders such as depression, anxiety, and mood disorders, as well as behavioral issues including attention deficit hyperactivity disorder (ADHD) and oppositional defiant disorder, with the latter negatively impacting social interactions when associated with antisocial behaviors [7]. The frequent misuse of smartphones and of the internet further exacerbates psychological health risks for adolescents by leading to negative impacts on self-esteem; sleep quality; increased anxiety, depression, aggressiveness; and difficulties in social relationships with peers and family [8]. These factors, along with higher a Body Mass Index (BMI) and dissatisfaction with body image, represent significant risk factors for eating disorders, especially among female adolescents [9].

During adolescence, the desire of independence along with psychological stress may lead to risky behaviors that increase the likelihood of diabetes-related complications. For instance, secondary diabetic ketoacidosis can occur in subjects with already known T1D who deliberately omit insulin administration and is frequently characterized by severe presentation [10]. Moreover, prolonged diabetes duration and suboptimal glycemic control can lead to early onset chronic complications such as retinopathy, nephropathy, peripheral neuropathy, and cardiovascular autonomic neuropathy, contributing to morbidity and mortality, and representing a substantial burden for caregivers [11].

Puberty and adolescence are characterized by rapid endocrinological changes that result in an increase in insulin resistance, exposing individuals with diabetes to glycemic decompensation and requiring frequent and dynamic adjustments of insulin therapy. Additionally, diabetes-related complications such as dermatological reactions to therapeutic tools can further negatively impact glycemic control and psychological well-being [12,13]. On the other hand, glucose homeostasis plays an important role in pubertal development, and conditions such as menarche delay and menstrual irregularities are frequently observed among youths with T1D [6].

Understanding behavioral and psychological issues of adolescents with T1D and their impact on glycemic control is crucial for optimizing diabetes management in this complex age group. The aim of this narrative review is to provide a comprehensive overview of the clinical and psychological features of T1D during adolescence, adding to the existing literature a complete summary of the main difficulties and their potential solution in T1D management over the course of adolescence. By reviewing the existing literature, we explored glucose control in adolescents with T1D, the psychological issues more commonly affecting this population, and targeted strategies to improve glycemic control.

## 2. Psychological Issues in Adolescents with T1D

The management of T1D during adolescence can present significant challenges in terms of psychological well-being. Although some studies suggest no clear evidence of increased psychosocial problems in adolescents with T1D [14], the current literature indicates an elevated risk of psychopathology in this population [6]. Higher rates of depression (10–26%), anxiety (9–19%), eating disorders (8–30%), behavioral disorders (12–20%), and substance abuse (25–50%) compared to the general population have been reported. Risk factors include demographic features such as female sex, diabetes-specific variables such as suboptimal glycemic control, and family functioning-related variables. Additional risk factors involve less frequent blood glucose monitoring, associated with poor treatment adherence. A family history of depression, particularly maternal depression, and diabetes-specific family conflicts, which can cause stress to parents or caregivers, can further reduce the psycho-emotional support provided by the family. These factors are significant predictors of psychological disorders in adolescents with T1D [15].

Anxiety and depression are among the most common mental disorders in young people with T1D [16]. According to a qualitative study conducted by Rechenberg et al., significant contributors to the onset of anxious symptoms include everyday stressors such as school and extracurricular activities, which can negatively impact sleep quality. The additional responsibilities related to diabetes management, requiring time and attention, can limit participation in normal daily activities, contributing to anxiety. Emotional factors such as fear and worry, including the fear of hypoglycemia or fear of judgment when performing self-management tasks in public, are identified by participants as diabetes-specific anxieties. These are distinct from general anxiety, which is associated with poor sleep quality and overall quality of life factors [17]. On the other hand, depressive symptoms appear to be linked to factors such as the lack of acceptance of the diabetes diagnosis, difficulties in managing the condition, perceived social isolation, uncertainty about the future, and fears related to long-term complications and traumatic experiences such as severe hypoglycemic episodes [18].

Adolescents with T1D, particularly females, are more vulnerable to eating disorders compared to their non-diabetic peers. Several factors contribute to the increased risk of eating disorders, including strict dietary control and the desire to maintain physical appearance, often leading to the deliberate omission of insulin for weight loss [19]. Recognizing eating disorders in people with diabetes can often be challenging. For instance, behaviors such as insulin omission for weight control may be mistakenly interpreted as poor diabetes management rather than indicators of an underlying eating disorder [20]. Family dynamics strongly influence unhealthy weight control behaviors and disordered eating. Family cohesion is inversely proportional to the likelihood of disordered eating behaviors, although no significant correlations exist with other aspects of the family environment, such as control, independence, and responsibility for diabetes management [21].

The complex management of diabetes also has implications within the school environment. Adolescents with T1D are more vulnerable to bullying, not only by peers but also by teachers, who may engage in discriminatory practices through verbal comments or situations of discomfort. Victimization by teachers can negatively impact the psychological well-being of youths, leading to anxiety, depression, and low self-esteem, as well as affecting academic performance by negatively influencing motivation and academic outcomes, creating a hostile and unwelcoming school environment [22]. Peer bullying can similarly impact mental health and self-esteem. The psychological stress and social isolation resulting from bullying can affect social relationships and adaptation, compromising the quality of life of teenagers with T1D [23].

Therefore, psychological care for adolescents with T1D is crucial. Cognitive behavioral therapy (CBT) has shown great efficacy in reducing anxiety and depression symptoms by helping young people with T1D identify and modify negative thoughts and dysfunctional beliefs [24]. CBT also provides adaptive coping strategies aimed at stress reduction [25]. This approach has also been proven to be effective in improving therapeutic adherence, as it enhances awareness and acceptance of the diabetes condition, leading to better glycemic control and more consistent insulin administration [26]. Furthermore, CBT helps reduce avoidance behaviors that can interfere with proper diabetes management by promoting the development of self-efficacy [27]. Support groups can also be particularly beneficial for young people with T1D and their families, allowing them to share similar experiences in diabetes management, improving parental responsibility, and enhancing quality of life [28].

## 3. Factors Influencing Glycemic Control in Adolescents with T1D

**Figure 1:**
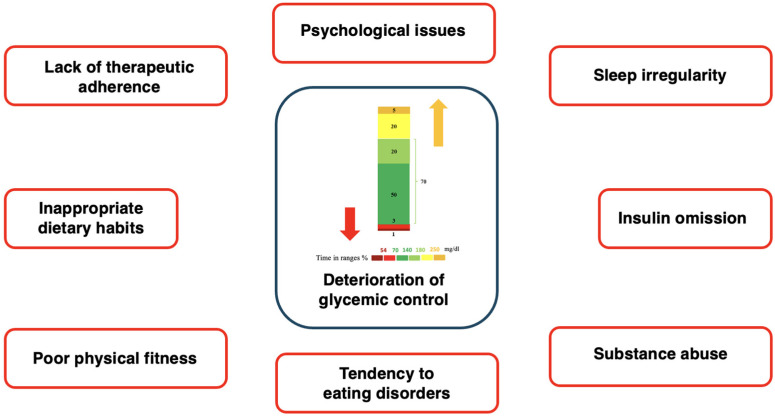
Graphical summary of factors influencing glycemic control in adolescents with T1D.

A deterioration of glycemic control is frequently observed during adolescence due to biological, psychological, and social factors that hinder optimal diabetes management (Figure 1).

Data from the Australasian Diabetes Data Network indicate suboptimal glycemic control among 6329 teenagers and young adults with T1D, with only 12.3% achieving the glycosylated hemoglobin (HbA1c) target of <7%. Higher HbA1c values were recorded among females and subjects with longer diabetes duration. Conversely, lower HbA1c values were associated with the attendance of pediatric diabetes facilities and with the use of insulin pumps [29]. Another longitudinal cohort study on 76 individuals with T1D aged 11–18 years revealed a progressive increase in HbA1c levels during adolescence, peaking at 18–19 years of age, alongside a parallel increase in overweight rates [30].

The lack of therapeutic adherence represents a frequent barrier to achieving satisfactory glucose control in adolescents with T1D. A retrospective cross-sectional study conducted by Sohayla et al. found that adolescents with T1D who checked their blood or interstitial glucose ≥ four times a day had lower HbA1c levels, while 97% of participants with inconsistent glucose monitoring did not meet the HbA1c threshold of 7% [31]. On the other hand, self-management skills along with the capacity of teenagers to complete tasks faster, as assessed by the Self-Report Behavioral Automaticity Index, have been shown to improve mean glucose levels in a cohort of 70 individuals with T1D [32].

Suboptimal glycemic control negatively affects several physiological processes typical of adolescence, including menstrual regularity in females. Schroeder et al. found that 19% of girls with T1D aged 10–18 years experienced menstrual disturbances, such as primary amenorrhea, secondary amenorrhea, and oligomenorrhea. The study also identified a strong association between higher HbA1c values and menstrual disturbances [33].

### 3.1. Dietary Habits

Dietary recommendations during adolescence should align with general healthy eating principles applicable to all young people. The goals are to maintain an ideal body weight in order to support optimal growth and prevent both acute and chronic complications [5].

Diet and macronutrient components significantly impact glycemic trends. A cross-sectional study comparing the carbohydrate intakes of 712 adolescents with T1D to a population of healthy individuals found that T1D subjects were more prone to consuming a higher weekly number of meals and snacks and that total carbohydrate intake was related to higher HbA1c values [34]. Similarly, a CGM analysis by Lejk et al. of a cohort of 26 adolescents found that a standard diet containing 50% carbohydrates was associated with higher glucose variability compared to a low-carbohydrate diet (30%) [35]. Myśliwiec et al., in a study on 20 male adolescents with T1D who were treated with insulin pumps, found that most participants followed an unbalanced diet characterized by excessive carbohydrate consumption [36].

Nevertheless, the intake of other macronutrients, beyond carbohydrates, also has a significant impact on postprandial glucose control. A randomized crossover trial involving 30 adolescents with T1D demonstrated that consumption of drinks high in fat led to a significant increase in glucose levels 4 to 8 h after ingestion, compared to drinks with lower fat content [37].

The mediterranean diet is strongly recommended for individuals with T1D due to its proven benefits on glycemic control and cardiovascular risk [5]. A prospective clinical intervention study analyzing the effects of switching to a mediterranean diet in 20 adolescents with T1D revealed, after the reduction in carbohydrate intake and the increase in monounsaturated fats, improvements in mean time in range from 52% to 63%, a decrease in total daily insulin dose, and positive effects on cardiovascular risk factors such as diastolic blood pressure and LDL cholesterol [38].

### 3.2. Physical Fitness

Physical exercise, by improving blood glucose levels, increasing insulin sensitivity, reducing cardiovascular risk factors, and enhancing the overall quality of life, is a cornerstone of T1D management [39]. However, adolescents with T1D often show reduced aerobic exercise capacity compared to their healthy peers. Komatsu et al. found that 72 adolescents with T1D had worse gas exchange variables and higher fatigability during a treadmill test compared to a control group of healthy subjects [40]. This contributes further to the difficulty to achieving glycemic recommended targets in this age group, as demonstrated by a Jordanian study revealing a significant association between suboptimal glycemic control and lower physical activity as expressed by metabolic equivalents per minute per week, with only 14.8% of teenage participants achieving an HbA1c value ≤ 7.5% [41]. Similarly, Qadir et al. demonstrated the HbA1c lowering effect of regular swimming on 40 male adolescents, with a significant drop of HbA1c values after 10 weeks of a training program [42]. Conversely, a multicenter cross-sectional study found only a weak association between exercise and metabolic control, and no relationship with BMI, hypoglycemia frequency, and diabetic ketoacidosis [43]. The implications of physical activity on cardiovascular health of people with T1D have been demonstrated since adolescence. An echocardiography-based study revealed well-preserved left ventricular systolic and diastolic function among adolescents with T1D with an adequate exercise capacity, even though ejection fraction values were worse in subjects with longer disease duration [44].

### 3.3. Sleep Characteristics

Sleep routines are closely related to glucose patterns in subjects with T1D. Adolescents often experience irregular sleep–wake rhythms due to common habits such as a nocturnal social life and the excessive use of electronic devices. Jaser et al., in a study assessing sleep quality of 159 adolescents through the Pittsburgh Sleep Quality Index, found an average sleep duration of 7.4 h, insufficient according to pediatric recommendations, and a correlation between lower sleep duration and worse glycemic control. Of note, individuals on insulin pump therapy had longer sleep duration [45]. Another study identified sleep duration as a modifiable factor influencing glycemic control, with poor sleep quality and sleep duration variability significantly related to HbA1c values, frequency of blood glucose monitoring, and average blood glucose levels [46]. Similarly, Schnurbein et al. confirmed the association between sleep duration and HbA1c, identifying circadian misalignment as a determinant of increased insulin requirements [47]. Furthermore, it has been proven that sleep quality is associated with glucose variability, recently identified as an independent risk factor for diabetes-related long-term complications [48,49].

### 3.4. Risky Behaviors and Substance Abuse

The management of T1D in adolescents is complicated by risky behaviors that may have dangerous consequences. Education and support, regular counseling, and psychosocial care and treatment to support the cessation of bad habits such as illicit drugs, alcohol, tobacco use, and vaping are crucial to achieving diabetes goals [39]. Recurrent hypoglycemia, as reported in a case series, may result from the voluntary administration of extra insulin, with the purpose of consuming more carbohydrates or seeking attention [50].

A survey-based study by Snyder et al. examining the prevalence of substance use, including alcohol, tobacco, illicit drugs, and insulin misuse among 60 adolescents with T1D, found that 36.7% had consumed substances, whereas insulin misuse was reported by 19%. Older participants and individuals with depression were more prone to substance use, while insulin misusers were mainly adolescents with disordered eating behaviors, with one-third reporting self-harm intent. However, no connection was found between glycemic control and these risky behaviors [51]. Conversely, a multicenter analysis on 29,630 subjects with T1D identified a notable association between alcohol consumption and worse glucose control assessed by HbA1c values, severe hypoglycemia and diabetic ketoacidosis [52]. Current evidence indicates that cannabis use among youths and young adults with T1D, accounting for around 10–30%, is associated with higher HbA1c values and increased diabetic ketoacidosis incidence, likely due to poorer self-management during episodes of use [53].

### 3.5. Mental Health

Daily difficulties faced by adolescents, exacerbated by diabetes, directly influence diabetes management, metabolic control, and HbA1c levels.

Anxiety plays a crucial role in metabolic control, beyond affecting cortisol levels in adolescents with T1D [54], by predisposing them to greater emotional distress through negative automatic thoughts and leading to a higher perception of stress and frustration related to diabetes [55]. Anxiety may also have a negative effect on therapeutic adherence due to fear of hypoglycemia, less frequent glucose monitoring, and limited coping strategies, directly impacting HbA1c levels [56].

Depressive symptoms can also have a significant impact on T1D management and glycemic control due to low motivation to follow prescribed therapies, such as regular glucose monitoring and insulin administration, resulting in increased HbA1c levels [57].

Eating disorders also affect metabolic control in adolescents with T1D. Disordered eating behaviors, such as an unbalanced and irregular diet or the practice of skipping or reducing insulin doses to control weight, are associated with higher HbA1c levels [21]. This practice, known as diabulimia, is linked to short- and long-term complications, including abnormal lipid profiles, diabetic ketoacidosis, retinopathy, neuropathy, nephropathy, and even higher mortality rates [58].

### 3.6. Social and School Environment

The social and school environment plays a fundamental role in diabetes management. Stress levels reported by adolescents with T1D who have been bullied are associated with long-term glycemic deterioration, and a relationship with the ability to manage diabetes autonomously and correctly follow the therapeutic regimen has been demonstrated [23]. Teacher victimization of students can lead to poor adherence to diabetes treatment through missed insulin administration or blood glucose monitoring, negatively affecting diabetes management at school [22]. Hegelson et al., in a 11-year cohort study involving 132 adolescents with T1D, found that parent social status, household structure, psychological distress, friend conflict, unmitigated communion, and self-care behavior significantly impacted HbA1c trajectories from early adolescence to adulthood [59].

## 4. Strategies to Improve Diabetes Management in Adolescents with T1D

Due to the presence of numerous factors hindering diabetes management and to the substantial risks associated with diabetes during adolescence, targeted interventions aimed at improving glycemic control are essential during this complex stage of life.

Healthcare education is an important measure for improving diabetes self-management and glycemic outcomes. Significant benefits of an educational intervention on glycemic control and quality of life of 503 adolescents with T1D were demonstrated by Abolfotouh et al. Improvements were observed in total knowledge, glucose monitoring, physical activity practice, treatment, self-efficacy, family involvement, glycemic control, and satisfaction among subjects undergoing the educational intervention [60].

During the last decades, technologies have radically improved the standards of diabetes care. The introduction of continuous glucose monitoring (CGM) systems in clinical practice, by providing real-time insights into glucose levels, have allowed more consistence in glucose monitoring, with particular advantages for adolescents with diabetes. Laffel et al., in a randomized clinical trial, demonstrated a beneficial effect of CGM use on a cohort of 153 adolescents and young adults with T1D, assessed by improved HbA1c [61].

Insulin pumps, through their feature of simulating physiological basal insulin secretion, have shown substantial benefits on glycemic outcomes compared to multiple daily injection therapy [62]. Patch pumps are tubeless devices that present an improved wearability compared to pumps with reservoir and, for this reason, are preferred by many adolescents. A prospective observational study showed increased treatment satisfaction score among adolescents using patch pumps compared to multiple daily injections therapy; however, no significant difference in HbA1c was detected [63].

The recent introduction of automated insulin delivery (AID) systems represents a game changer in T1D management, offering notably improved performances on glycemic control compared to other treatment modalities and protection against hypoglycemia [64,65]. Isganaitis et al. demonstrated the efficacy and safety of AID systems in a randomized controlled trial involving adolescents and young adults with T1D, showing better time in range and reduced hypoglycemia rates after 6 months of use compared to sensor-augmented pump systems [66]. The adoption of commands input via smartphone apps to manage meal-time boluses and to adjust insulin delivery may further enhance the usability and acceptability of AID systems among adolescents, as shown by a qualitative study on teenager using the CamAPS system [67]. Timely and accurate carbohydrate counting is required by AID systems currently available on the market. However, many adolescents may encounter difficulties in performing this task, with consequent bolus omission or underestimation. Adherence to insulin boluses at mealtime has a great impact on glycemic control, as demonstrated by a study on 90 teenagers, showing that better adherence was associated with lower HbA1c values [68]. The importance of accurate carb counting has also been highlighted by Petrovski et al. in a clinical trial involving 34 adolescents with T1D using a second-generation AID system, demonstrating better glycemic outcomes among subjects performing precise carbohydrate counting compared to individuals using a simplified meal announcement [69].

Another strategy that can be adopted is telemedical support, which has been proven to be effective in improving glycemic control of people with diabetes. Rami et al., in a crossover trial, evaluated the feasibility and the benefits of a telemedical support program in adolescents with T1D, showing improved HbA1c levels during the intervention, with prompt deterioration of glucose control after switching back to conventional support [70].

Several studies indicate that diabetes camps can bring benefits on glycemic control by improving knowledge and independence in T1D management. A retrospective study evaluating glycemic control of a cohort adolescents with T1D attending a diabetes camp showed a decrease in HbA1c levels during the follow-up, with sustained benefits on glucose control after 7 month and improvements on parental-reported adherence [71]. Additionally, Santiprabhob et al. reported psychological advantages from attending a 5-day diabetes camp in a group of 60 individuals. After a 6-month follow-up period, improvements in HbA1c levels, self-reported knowledge about diabetes, and coping mechanisms were observed [72].

Shared medical appointments represent another approach that could possibly contain glycemic control decline during adolescence. A study involving 37 subjects with T1D undergoing three shared medical appointments has shown improvements in quality of life, school and psychosocial functions, and adherence and communication skills, with a reduction in barriers. However, no significant changes in HbA1c have been detected during the study period [73].

As a possible strategy to improve treatment adherence, Wong et al. investigated the effect of daily financial incentives on glucose monitoring and glycemic control in 90 adolescents with T1D and suboptimal glucose control, showing greater adherence but no significant change in HbA1c [74]. A randomized controlled trial involving 92 subjects investigated the usefulness of daily text message reminders at the time of insulin injections in overcoming the lack of treatment adherence of adolescents with T1D with poor baseline glycemic control. A significant decrease in HbA1c values after 6 months of text message intervention was observed, with high self-reported satisfaction [75].

## 5. Conclusions

Managing T1D during adolescence may be often challenging due to the physical, psychological, and behavioral changes, specific to this developmental stage, which may interfere with effective diabetes management. Given the particular vulnerability to psychological issues, it is imperative to implement targeted interventions to support adolescents facing mental health challenges, such as anxiety, depression, and eating disorders. These interventions are crucial not only for preventing or managing psychological comorbidities associated with diabetes but also because these conditions significantly impact diabetes management. Comprehensive psychological assessments and appropriate interventions can therefore benefit both the mental and physical health of adolescents with T1D.

To optimize treatment adherence in this population, strategies should focus on encouraging a healthy lifestyle, including balanced nutrition, regular physical activity, and proper sleep hygiene. Recent advancements in diabetes technology have made recommended glycemic targets more attainable for youths with T1D. Consequently, the use of CGM and AID systems should be strongly encouraged in this population. Continuous health education is also crucial to increase awareness about the condition and to discourage harmful behaviors such as substances abuse.

## Data Availability

No new data were created or analyzed in this study.

## References

[1] Atkinson M.A., Eisenbarth G.S., Michels A.W. (2014). Type 1 diabetes. Lancet.

[2] Gregory G.A., Robinson T.I.G., Linklater S.E., Wang F., Colagiuri S., de Beaufort C., Donaghue K.C., Magliano D.J., Maniam J., International Diabetes Federation Diabetes Atlas Type 1 Diabetes in Adults Special Interest Group (2022). Global incidence, prevalence, and mortality of type 1 diabetes in 2021 with projection to 2040: A modelling study. Lancet Diabetes Endocrinol..

[3] Cengiz E., Danne T., Ahmad T., Ayyavoo A., Beran D., Ehtisham S., Fairchild J., Jarosz-Chobot P., Ng S.M., Paterson M. (2022). ISPAD Clinical Practice Consensus Guidelines 2022: Insulin treatment in children and adolescents with diabetes. Pediatr. Diabetes.

[4] Adolfsson P., Taplin C.E., Zaharieva D.P., Pemberton J., Davis E.A., Riddell M.C., McGavock J., Moser O., Szadkowska A., Lopez P. (2022). ISPAD Clinical Practice Consensus Guidelines 2022: Exercise in children and adolescents with diabetes. Pediatr. Diabetes.

[5] Annan S.F., Higgins L.A., Jelleryd E., Hannon T., Rose S., Salis S., Baptista J., Chinchilla P., Marcovecchio M.L. (2022). ISPAD Clinical Practice Consensus Guidelines 2022: Nutritional management in children and adolescents with diabetes. Pediatr. Diabetes.

[6] Gregory J.W., Cameron F.J., Joshi K., Eiswirth M., Garrett C., Garvey K., Agarwal S., Codner E. (2022). ISPAD Clinical Practice Consensus Guidelines 2022: Diabetes in adolescence. Pediatr. Diabetes.

[7] Angold A., Costello E.J. (1995). Developmental epidemiology. Epidemiol. Rev..

[8] Lissak G. (2018). Adverse physiological and psychological effects of screen time on children and adolescents: Literature review and case study. Environ. Res..

[9] Suarez-Albor C.L., Galletta M., Gómez-Bustamante E.M. (2022). Factors associated with eating disorders in adolescents: A systematic review. Acta Bio-Medica Atenei Parm..

[10] Passanisi S., Salzano G., Basile P., Bombaci B., Caime F., Rulli I., Valenzise M., Gitto E., Lombardo F. (2023). Prevalence and clinical features of severe diabetic ketoacidosis treated in pediatric intensive care unit: A 5-year monocentric experience. Ital. J. Pediatr..

[11] Gomes M.B., Calliari L.E., Conte D., Correa C.L., Drummond K.R.G., Mallmann F., Pinheiro A.A., Muniz L.H., Leal F.S.L., Morales P.H. (2021). Diabetes-related chronic complications in Brazilian adolescents with type 1 diabetes. A multicenter cross-sectional study. Diabetes Res. Clin. Pract..

[12] Passanisi S., Galletta F., Bombaci B., Cherubini V., Tiberi V., Minuto N., Bassi M., Iafusco D., Piscopo A., Mozzillo E. (2024). Device-Related Skin Reactions Increase Emotional Burden in Youths With Type 1 Diabetes and Their Parents. J. Diabetes Sci. Technol..

[13] Ledwoń E., Zemła-Szten P., von dem Berge T., Nalewajko K., Passanisi S., Piona C., Dos Santos T.J., Svensson J., Korsgaard Berg A., Chobot A. (2024). Skin Reactions in Children with Type 1 Diabetes Associated with the Use of New Diabetes Technologies-An Observational Study from a Regional Polish Pediatric Diabetes Center. Children.

[14] Sivertsen B., Petrie K.J., Wilhelmsen-Langeland A., Hysing M. (2014). Mental health in adolescents with Type 1 diabetes: Results from a large population-based study. BMC Endocr. Disord..

[15] Kakleas K., Kandyla B., Karayianni C., Karavanaki K. (2009). Psychosocial problems in adolescents with type 1 diabetes mellitus. Diabetes Metab..

[16] Watson S.E., Spurling S.E., Fieldhouse A.M., Montgomery V.L., Wintergerst K.A. (2020). Depression and Anxiety Screening in Adolescents With Diabetes. Clin. Pediatr..

[17] Rechenberg K., Grey M., Sadler L. (2018). “Anxiety and Type 1 diabetes are like cousins”: The experience of anxiety symptoms in youth with Type 1 diabetes. Res. Nurs. Health.

[18] Buchberger B., Huppertz H., Krabbe L., Lux B., Mattivi J.T., Siafarikas A. (2016). Symptoms of depression and anxiety in youth with type 1 diabetes: A systematic review and meta-analysis. Psychoneuroendocrinology.

[19] Jones J.M., Lawson M.L., Daneman D., Olmsted M.P., Rodin G. (2000). Eating disorders in adolescent females with and without type 1 diabetes: Cross sectional study. BMJ.

[20] Peveler R.C., Fairburn C.G., Boller I., Dunger D. (1992). Eating disorders in adolescents with IDDM. A controlled study. Diabetes Care.

[21] Neumark-Sztainer D., Patterson J., Mellin A., Ackard D.M., Utter J., Story M., Sockalosky J. (2002). Weight control practices and disordered eating behaviors among adolescent females and males with type 1 diabetes: Associations with sociodemographics, weight concerns, familial factors, and metabolic outcomes. Diabetes Care.

[22] Peters C.D., Storch E.A., Geffken G.R., Heidgerken A.D., Silverstein J.H. (2008). Victimization of youth with type-1 diabetes by teachers: Relations with adherence and metabolic control. J. Child. Health Care Prof. Work. Child. Hosp. Community.

[23] do Nascimento Andrade C.J., de Aragão Dantas Alves C. (2019). Relationship between bullying and type 1 diabetes mellitus in children and adolescents: A systematic review. J. Pediatr..

[24] Wei C., Allen R.J., Tallis P.M., Ryan F.J., Hunt L.P., Shield J.P., Crowne E.C. (2018). Cognitive behavioural therapy stabilises glycaemic control in adolescents with type 1 diabetes-Outcomes from a randomised control trial. Pediatr. Diabetes.

[25] Grey M., Boland E.A., Davidson M., Li J., Tamborlane W.V. (2000). Coping skills training for youth with diabetes mellitus has long-lasting effects on metabolic control and quality of life. J. Pediatr..

[26] Wysocki T., Greco P., Harris M.A., Bubb J., White N.H. (2001). Behavior therapy for families of adolescents with diabetes: Maintenance of treatment effects. Diabetes Care.

[27] Hood K.K., Beavers D.P., Yi-Frazier J., Bell R., Dabelea D., Mckeown R.E., Lawrence J.M. (2014). Psychosocial burden and glycemic control during the first 6 years of diabetes: Results from the SEARCH for Diabetes in Youth study. J. Adolesc. Health.

[28] Kichler J.C., Kaugars A.S., Marik P., Nabors L., Alemzadeh R. (2013). Effectiveness of groups for adolescents with type 1 diabetes mellitus and their parents. Fam. Syst. Health J. Collab. Fam. Healthc..

[29] James S., Perry L., Lowe J., Harris M., Craig M.E., ADDN study group (2022). Suboptimal glycemic control in adolescents and young adults with type 1 diabetes from 2011 to 2020 across Australia and New Zealand: Data from the Australasian Diabetes Data Network registry. Pediatr. Diabetes.

[30] Bryden K.S., Peveler R.C., Stein A., Neil A., Mayou R.A., Dunger D.B. (2001). Clinical and psychological course of diabetes from adolescence to young adulthood: A longitudinal cohort study. Diabetes Care.

[31] Ibrahim S.A., El Hajj M.S., Owusu Y.B., Al-Khaja M., Khalifa A., Ahmed D., Awaisu A. (2022). Adherence as a Predictor of Glycemic Control Among Adolescents With Type 1 Diabetes: A Retrospective Study Using Real-world Evidence. Clin. Ther..

[32] Cummings C., Benjamin N.E., Prabhu H.Y., Cohen L.B., Goddard B.J., Kaugars A.S., Humiston T., Lansing A.H. (2022). Habit and diabetes self-management in adolescents with type 1 diabetes. Health Psychol. Off. J. Div. Health Psychol. Am. Psychol. Assoc..

[33] Schroeder B., Hertweck S.P., Sanfilippo J.S., Foster M.B. (2000). Correlation between glycemic control and menstruation in diabetic adolescents. J. Reprod. Med..

[34] Baechle C., Hoyer A., Castillo-Reinado K., Stahl-Pehe A., Kuss O., Holl R.W., Kersting M., Rosenbauer J., In cooperation with the German Pediatric Surveillance Unit (ESPED) and the DPV-Science initiative, supported by the German Center for Diabetes Research (DZD) (2018). Eating Frequency and Carbohydrate Intake in Adolescents with Type 1 Diabetes Differ from Those in Their Peers and are Associated with Glycemic Control. Exp. Clin. Endocrinol. Diabetes Off. J. Ger. Soc. Endocrinol. Ger. Diabetes Assoc..

[35] Lejk A., Chrzanowski J., Cieślak A., Fendler W., Myśliwiec M. (2022). Reduced Carbohydrate Diet Influence on Postprandial Glycemia-Results of a Short, CGM-Based, Interventional Study in Adolescents with Type 1 Diabetes. Nutrients.

[36] Myśliwiec A., Lejk A., Skalska M., Jastrzębska J., Sztangierska B., Jastrzębski Z. (2021). Assessment of the diet of male adolescents suffering from type 1 diabetes. Pediatr. Endocrinol. Diabetes Metab..

[37] O’Connell S.M., O’Toole N.M.A., Cronin C.N., Saat-Murphy C., McElduff P., King B.R., Smart C.E., Shafat A. (2021). Does dietary fat cause a dose dependent glycemic response in youth with type 1 diabetes?. Pediatr. Diabetes.

[38] Levran N., Levek N., Sher B., Mauda-Yitzhak E., Gruber N., Afek A., Monsonego-Ornan E., Pinhas-Hamiel O. (2023). The Mediterranean Diet for Adolescents with Type 1 Diabetes: A Prospective Interventional Study. Nutrients.

[39] American Diabetes Association Professional Practice Committee 5 (2024). Facilitating Positive Health Behaviors and Well-being to Improve Health Outcomes: Standards of Care in Diabetes-2024. Diabetes Care.

[40] Komatsu W.R., Gabbay M.A.L., Castro M.L., Saraiva G.L., Chacra A.R., de Barros Neto T.L., Dib S.A. (2005). Aerobic exercise capacity in normal adolescents and those with type 1 diabetes mellitus. Pediatr. Diabetes.

[41] Aljawarneh Y.M., Wood G.L., Wardell D.W., Al-Jarrah M.D. (2023). The associations between physical activity, health-related quality of life, regimen adherence, and glycemic control in adolescents with type 1 diabetes: A cross-sectional study. Prim. Care Diabetes.

[42] Qadir K.J., Zangana K.O. (2020). Effect of swimming program on glycemic control in male adolescents with type 1 diabetes mellitus. J. Sports Med. Phys. Fit..

[43] Aman J., Skinner T.C., de Beaufort C.E., Swift P.G.F., Aanstoot H.-J., Cameron F., Hvidoere Study Group on Childhood Diabetes (2009). Associations between physical activity, sedentary behavior, and glycemic control in a large cohort of adolescents with type 1 diabetes: The Hvidoere Study Group on Childhood Diabetes. Pediatr. Diabetes.

[44] Van Ryckeghem L., Franssen W.M.A., Verbaanderd E., Indesteege J., De Vriendt F., Verwerft J., Dendale P., Bito V., Hansen D. (2021). Cardiac Function is Preserved in Adolescents With Well-Controlled Type 1 Diabetes and a Normal Physical Fitness: A Cross-Sectional Study. Can. J. Diabetes.

[45] Jaser S.S., Ellis D. (2016). Sleep in Adolescents and Young Adults with Type 1 Diabetes: Associations with Diabetes Management and Glycemic Control. Health Psychol. Behav. Med..

[46] Patel N.J., Savin K.L., Kahanda S.N., Malow B.A., Williams L.A., Lochbihler G., Jaser S.S. (2018). Sleep habits in adolescents with type 1 diabetes: Variability in sleep duration linked with glycemic control. Pediatr. Diabetes.

[47] von Schnurbein J., Boettcher C., Brandt S., Karges B., Dunstheimer D., Galler A., Denzer C., Denzer F., Vollbach H., Wabitsch M. (2018). Sleep and glycemic control in adolescents with type 1 diabetes. Pediatr. Diabetes.

[48] Griggs S., Redeker N.S., Jeon S., Grey M. (2020). Daily variations in sleep and glucose in adolescents with type 1 diabetes. Pediatr. Diabetes.

[49] Bombaci B., Passanisi S., Lombardo F., Salzano G. (2024). Clinical relevance of short-term glycemic variability in children and adolescents with type 1 diabetes: A narrative review. Transl. Pediatr..

[50] Jain V., Satapathy A.K., Yadav J. (2015). Surreptitious insulin overdosing in adolescents with type 1 diabetes. Indian Pediatr..

[51] Snyder L.L., Truong Y.K.-N., Law J.R. (2016). Evaluating Substance Use and Insulin Misuse in Adolescents With Type 1 Diabetes. Diabetes Educ..

[52] Hermann J.M., Meusers M., Bachran R., Kuhnle-Krahl U., Jorch N., Hofer S.E., Holl R.W., DPV initiative (2017). Self-reported regular alcohol consumption in adolescents and emerging adults with type 1 diabetes: A neglected risk factor for diabetic ketoacidosis? Multicenter analysis of 29 630 patients from the DPV registry. Pediatr. Diabetes.

[53] Pancer J., Dasgupta K. (2020). Effects of Cannabis Use in Youth and Young Adults With Type 1 Diabetes: The Highs, the Lows, the Don’t Knows. Can. J. Diabetes.

[54] El Mlili N., Ahabrach H., Bahri H., Kerkeb A., Mafla-España M.A., Cauli O. (2023). Psychological Alterations in Youths with Type I Diabetes: Associations with Salivary Cortisol Concentration. Medicina.

[55] Vesco A.T., Howard K.R., Anderson L.M., Papadakis J.L., Hood K.K., Weissberg-Benchell J. (2021). Examining Indirect Effects of Anxiety on Glycated Hemoglobin via Automatic Negative Thinking and Diabetes-Specific Distress in Adolescents With Type 1 Diabetes. Can. J. Diabetes.

[56] Rechenberg K., Whittemore R., Grey M. (2017). Anxiety in Youth With Type 1 Diabetes. J. Pediatr. Nurs..

[57] Hood K.K., Huestis S., Maher A., Butler D., Volkening L., Laffel L.M.B. (2006). Depressive symptoms in children and adolescents with type 1 diabetes: Association with diabetes-specific characteristics. Diabetes Care.

[58] Colton P.A., Olmsted M.P., Daneman D., Farquhar J.C., Wong H., Muskat S., Rodin G.M. (2015). Eating Disorders in Girls and Women With Type 1 Diabetes: A Longitudinal Study of Prevalence, Onset, Remission, and Recurrence. Diabetes Care.

[59] Helgeson V.S., Vaughn A.K., Seltman H., Orchard T., Libman I., Becker D. (2018). Featured Article: Trajectories of Glycemic Control Over Adolescence and Emerging Adulthood: An 11-Year Longitudinal Study of Youth With Type 1 Diabetes. J. Pediatr. Psychol..

[60] Abolfotouh M.A., Kamal M.M., El-Bourgy M.D., Mohamed S.G. (2011). Quality of life and glycemic control in adolescents with type 1 diabetes and the impact of an education intervention. Int. J. Gen. Med..

[61] Laffel L.M., Kanapka L.G., Beck R.W., Bergamo K., Clements M.A., Criego A., DeSalvo D.J., Goland R., Hood K., Liljenquist D. (2020). Effect of Continuous Glucose Monitoring on Glycemic Control in Adolescents and Young Adults With Type 1 Diabetes: A Randomized Clinical Trial. JAMA.

[62] Dos Santos T.J., Donado Campos J.d.M., Argente J., Rodríguez-Artalejo F. (2021). Effectiveness and equity of continuous subcutaneous insulin infusions in pediatric type 1 diabetes: A systematic review and meta-analysis of the literature. Diabetes Res. Clin. Pract..

[63] Nivet E., Lo G., Nivot-Adamiak S., Guitteny M.-A., De Kerdanet M. (2022). Impact of OMNIPOD^®^ on the quality of life of adolescents with type 1 diabetes. Arch. Pediatr. Organe Off. Soc. Fr. Pediatr..

[64] Bombaci B., Passanisi S., Alibrandi A., D’Arrigo G., Patroniti S., Averna S., Salzano G., Lombardo F. (2022). One-Year Real-World Study on Comparison among Different Continuous Subcutaneous Insulin Infusion Devices for the Management of Pediatric Patients with Type 1 Diabetes: The Supremacy of Hybrid Closed-Loop Systems. Int. J. Environ. Res. Public Health.

[65] Ferrito L., Passanisi S., Bonfanti R., Cherubini V., Minuto N., Schiaffini R., Scaramuzza A. (2021). Efficacy of advanced hybrid closed loop systems for the management of type 1 diabetes in children. Minerva Pediatr..

[66] Isganaitis E., Raghinaru D., Ambler-Osborn L., Pinsker J.E., Buckingham B.A., Wadwa R.P., Ekhlaspour L., Kudva Y.C., Levy C.J., Forlenza G.P. (2021). Closed-Loop Insulin Therapy Improves Glycemic Control in Adolescents and Young Adults: Outcomes from the International Diabetes Closed-Loop Trial. Diabetes Technol. Ther..

[67] Rankin D., Kimbell B., Allen J.M., Besser R.E.J., Boughton C.K., Campbell F., Elleri D., Fuchs J., Ghatak A., Randell T. (2021). Adolescents’ Experiences of Using a Smartphone Application Hosting a Closed-loop Algorithm to Manage Type 1 Diabetes in Everyday Life: Qualitative Study. J. Diabetes Sci. Technol..

[68] Spaans E., van Hateren K.J.J., Groenier K.H., Bilo H.J.G., Kleefstra N., Brand P.L.P. (2018). Mealtime insulin bolus adherence and glycemic control in adolescents on insulin pump therapy. Eur. J. Pediatr..

[69] Petrovski G., Campbell J., Pasha M., Day E., Hussain K., Khalifa A., van den Heuvel T. (2023). Simplified Meal Announcement Versus Precise Carbohydrate Counting in Adolescents With Type 1 Diabetes Using the MiniMed 780G Advanced Hybrid Closed Loop System: A Randomized Controlled Trial Comparing Glucose Control. Diabetes Care.

[70] Rami B., Popow C., Horn W., Waldhoer T., Schober E. (2006). Telemedical support to improve glycemic control in adolescents with type 1 diabetes mellitus. Eur. J. Pediatr..

[71] Wang Y.-C.A., Stewart S., Tuli E., White P. (2008). Improved glycemic control in adolescents with type 1 diabetes mellitus who attend diabetes camp. Pediatr. Diabetes.

[72] Santiprabhob J., Likitmaskul S., Kiattisakthavee P., Weerakulwattana P., Chaichanwattanakul K., Nakavachara P., Peerapatdit T., Nitiyanant W. (2008). Glycemic control and the psychosocial benefits gained by patients with type 1 diabetes mellitus attending the diabetes camp. Patient Educ. Couns..

[73] Floyd B.D., Block J.M., Buckingham B.B., Ly T., Foster N., Wright R., Mueller C.L., Hood K.K., Shah A.C. (2017). Stabilization of glycemic control and improved quality of life using a shared medical appointment model in adolescents with type 1 diabetes in suboptimal control. Pediatr. Diabetes.

[74] Wong C.A., Miller V.A., Murphy K., Small D., Ford C.A., Willi S.M., Feingold J., Morris A., Ha Y.P., Zhu J. (2017). Effect of Financial Incentives on Glucose Monitoring Adherence and Glycemic Control Among Adolescents and Young Adults With Type 1 Diabetes: A Randomized Clinical Trial. JAMA Pediatr..

[75] Ibrahim N., Treluyer J.-M., Briand N., Godot C., Polak M., Beltrand J. (2021). Text message reminders for adolescents with poorly controlled type 1 diabetes: A randomized controlled trial. PLoS ONE.

